# Subacute Subdural Hematoma in a Patient with Bilateral DBS Electrodes

**DOI:** 10.1155/2015/390727

**Published:** 2015-12-08

**Authors:** Ha Son Nguyen, Peter A. Pahapill

**Affiliations:** Department of Neurosurgery, Medical College of Wisconsin, 9200 Wisconsin Avenue, Milwaukee, WI 53226, USA

## Abstract

Subdural hematomas (SDH) in patients with implanted deep brain stimulating (DBS) electrodes are rare. Only a handful of cases have been reported in the literature. No clear management guidelines exist regarding the management of the hematoma and the existing electrodes. We describe a 68-year-old female with bilateral DBS electrodes, who presented with acute, severe hemiparesis due to a large subacute SDH with associated electrode displacement. Urgent hematoma evacuation reversed the hemiparesis; the electrodes were left undisturbed. Brain reexpansion occurred promptly. The patient was able to benefit from stable DBS therapies within 3 weeks of hematoma evacuation, maintained at 1.5-year follow-up. The case highlights that despite relative electrode migration due to a subdural hematoma, the electrodes may not require revision during initial hematoma evacuation or in a delayed fashion. Timely hematoma evacuation, coupled with brain reexpansion, may be adequate for the electrode to travel back to its original position and effect reasonable DBS therapies.

## 1. Introduction

The literature is scarce regarding the management of subdural hematoma (SDH) in patients with existing DBS systems. Subacute or chronic SDH is a rare complication after DBS lead implantation [1, 2]. Moreover, patients with preexisting DBS leads may incur an acute SDH following a traumatic brain injury [3, 4]. Reported management options advocate initial treatment of the subdural hematoma if clinically warranted without DBS hardware revision [1, 3–7]. Removal/revision of the DBS leads occurred in instances that involved infection [7]. Though subdural hematomas have been documented in DBS patients, the management and clinical outcomes have only been detailed in a few patients [3–5]. In this paper, the authors plan to report a DBS patient with a subacute subdural hematoma, while reviewing the literature and exploring management options.

## 2. Case Presentation

**Figure 1:**
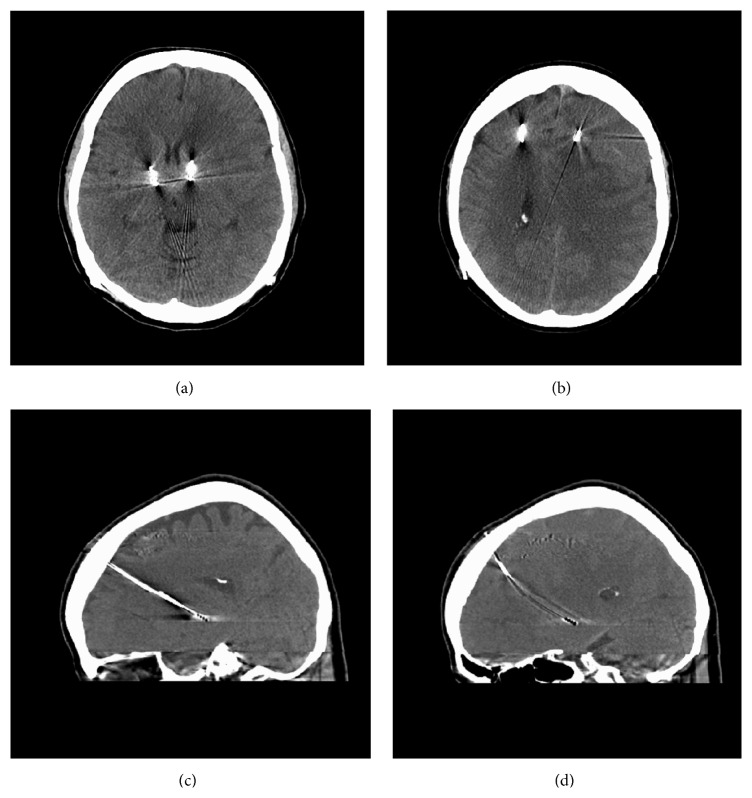
(a) CT head demonstrates left to right brain shift. (b) CT head demonstrates isodense pathology along left convexity. (c) CT head sagittal view demonstrates R DBS lead with relative normal position. (d) CT head sagittal view demonstrates L DBS lead that has bowed compared to the R DBS lead.

**Figure 2:**
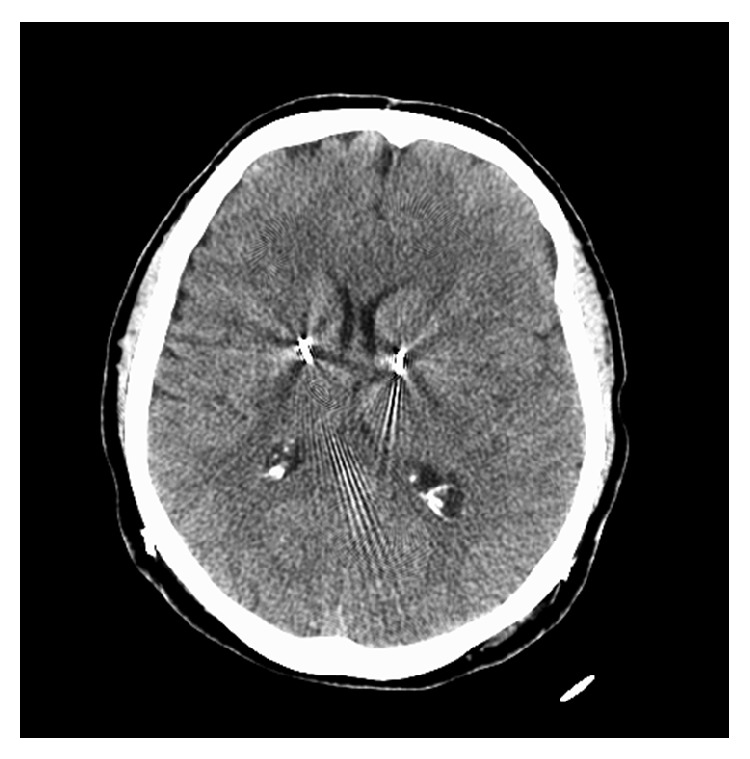
CT head at follow-up visit demonstrates resolution of SDH.

A 68-year-old female, with staged bilateral subthalamic nucleus (STN) leads and batteries, presented 36 days after placement of her left STN electrode. Patient noted left side dyskinesias and right side “off” symptoms. Her gait had a decent step out with the left foot but remained shuffled; on the right, she demonstrated a short stride with no right arm swing. Adjustments were beneficial for the right IPG, utilizing the interleaving mode, with program (1) C(+)1(−), 0.5 v, 60 uS, 125 Hz; program (2) C(+)2(−), 2.0 v, 60 uS, 125 Hz. The left IPG was reprogrammed with several different strategies with no major improvement in right-sided signs. Over a period of 48 hours, her right extremities became profoundly paretic with graded 1/5 strength. There was no history of trauma or antiplatelet/anticoagulant usage. She had no headaches, fever, nausea, vomiting, or altered sensorium. A head CT demonstrated an isodense left subacute SDH with a midline shift of 8 mm (Figures 1(a) and 1(b)). The left lead had bowed anteroinferiorly compared to the right lead (Figures 1(c) and 1(d)). Subsequently, the patient underwent urgent left burr-hole drainage of the SDH and placement of a subdural drain. Postoperative CT scans demonstrated progressive resolution of the hematoma (Figure 2), and the subdural drain was removed several days later. At the time of discharge on postoperative day 6, the patient had regained symmetric baseline strength with only a subtle right arm drift. DBS therapy was restored within 3 weeks after clot evacuation. Her baseline UPDRS Part III off-medication/on-stimulation right DBS was 48, and on-medication/on-stimulation was 10. After hematoma evacuation, no formal UPDRS evaluation was set up. However, throughout her postoperative course (up to 18 months), her movement neurologist noted that she continued to benefit from DBS stimulation with symmetrical stimulator settings while exhibiting no residual weakness. She noted minimal dyskinesia. She still experienced end-of-dose deterioration noted by start hesitation and gait freezing, usually at 3-4 hours after the dose. When she was “on,” she did fairly well. Medications lasted 3-4 hours depending on her activity level and lasted longer if she was more sedentary.

## 3. Discussion

DBS has gained prominence for the treatment of Parkinson's disease, essential tremors, and primary generalized dystonia. The efficacy of DBS relies on the precision of the lead placement. In 7 patients who demonstrated suboptimal response from DBS stimulation, Anheim et al. [8] reported that reimplantation of their leads, which improved the mean distance between contacts used for chronic stimulation and the theoretical effective target from 5.4 mm to 2.0 mm, resulted in better motor scores. Sources of error include distortion of the imaging used for targeting or from mechanical issues involving the frame system. Brain tissue distortion is another source of error and is an umbrella term for such pathologies like subdural hematoma, intraparenchymal hemorrhage, or pneumocephalus. Pneumocephalus has been discussed extensively due to its relatively frequent occurrence on postoperative imaging. Less has been discussed regarding subdural hematoma in DBS patients, likely due to its low incidence of less than 1% [5]. Review of the literature only uncovers a handful of DBS patients that have been followed long term after sustaining a subdural hematoma.

Oyama et al. [5] reported 4 patients that developed chronic subdural hematoma after DBS implantation for Parkinson's disease. There was no history of trauma. No patients required surgery acutely, while three patients required burr hole drainage eventually. Thresholds for stimulation-induced side effects were lower during the initial postoperative programming. No DBS hardware was revised. The authors noted that effective DBS therapy could still be achieved following reduction of subdural hematoma, but only after a significant delay of weeks to months (up to 18 months).

In addition, the conservative approach has also been applied for patients with acute subdural hematoma after a trauma. Yang et al. [4] reported a patient, with bilateral DBS leads for Parkinson's disease, who sustained a left acute subdural hematoma after a fall and underwent emergent craniectomy for hematoma evacuation without DBS hardware revision. The patient remained heavily dependent on the ventilator, which was partly attributed to poor control of her Parkinson's disease. On postoperative day 16, the DBS system was turned back on, and patient was extubated 3 days afterwards. The successful extubation was attributed to the resumption of her DBS system and better control of her Parkinson's disease. Park et al. [3] reported two patients with bilateral DBS leads, who sustained traumatic brain injury. One patient, who had bilateral thalamic leads for tremors, sustained an acute epidural hematoma. Another patient, who had bilateral cingulotomy and bilateral leads at CM-pf for Tourette's syndrome, suffered from an acute subdural hematoma. Both had emergent craniectomy for hematoma evacuation while preserving their DBS system. Both demonstrated benefits with DBS stimulation after hematoma evacuation. The first patient had a Fahn-Tolosa-Marin rating scale at 76 prior to lead placement, at 19 after one year postoperatively, and at 38 one year after the epidural evacuation. The second patient had a Yale Global Tic Severity Scale of 100 and Yale Brown Obsessive Compulsive Scale of 30 prior to DBS surgery; 6 and 5, respectively, after DBS surgery; and 70 and 18, respectively, after hematoma evacuation.

With a subdural hematoma, the brain will shift ventromedially; since DBS leads are tethered to the skull, the ipsilateral lead will have a relative dorsal displacement from its original target site [5]. Yang et al. [4] commented that gliosis formed along the track of the electrodes may function as a potential space. As a result, once the subdural hematoma is evacuated, the migrated electrode may glide back to its original location. Consequently, the DBS stimulation can remain effective without DBS revision surgery.

The etiology of the subacute SDH in the case presented here was likely related to the recent left DBS electrode implantation, presenting at 36 days, similar to the patients presented by Oyama et al. [5] (19–29 days). On the other hand, our patient exhibited a more dramatic clinical presentation with rapid, severe hemiparesis as compared to those of Oyama et al. [5]; yet the goals of hematoma evacuation without electrode revision were still achieved. Our patient obtained rapid reversal of neurological deficits and restoration of DBS therapies within 3 weeks of clot evacuation that have been sustained at 18 months follow-up with results similar to those of Oyamma et al. [5]. The case highlights that despite relative electrode migration due to a subdural hematoma, the electrodes may not require revision during initial hematoma evacuation or in a delayed fashion. Timely hematoma evacuation, coupled with brain reexpansion, may be adequate for the electrode to travel back to its original position and effect reasonable DBS therapies. Further research can focus on the reasoning behind a delayed response to DBS therapy, despite prompt resolution of the hematoma and complete reexpansion of brain parenchyma.

## 4. Conclusion

There are no established guidelines for the management of subdural hematoma in patients with DBS implantation. Reported management options advocate initial treatment of the subdural hematoma if clinically warranted without DBS hardware revision. This case highlights that despite relative electrode migration due to a subdural hematoma, the electrodes may not require revision during initial hematoma evacuation or in a delayed fashion. Timely hematoma evacuation, coupled with brain reexpansion, may be adequate for the electrode to travel back to its original position and effect reasonable DBS therapies. Further research can focus on the reasoning behind a delayed response to DBS therapy, despite prompt resolution of the hematoma and complete reexpansion of brain parenchyma.
